# Comprehensive transcriptome analysis of AP2/ERFs in *Osmanthus fragrans* reveals the role of *OfERF017*-mediated organic acid metabolism pathway in flower senescence

**DOI:** 10.3389/fpls.2024.1467232

**Published:** 2024-09-26

**Authors:** Jing-Jing Zou, Jun Zhang, Xiaoqian Wang, Hui Xia, Xiangling Zeng, Xuan Cai, Jie Yang, Jin Zeng, Zeqing Li, Guifu Zhang, Hongguo Chen

**Affiliations:** ^1^ National Forestry and Grassland Administration Engineering Research Center for Osmanthus fragrans, Hubei University of Science and Technology, Xianning, China; ^2^ Osmanthus Innovation Center of National Engineering Research Center for Floriculture, Hubei University of Science and Technology, Xianning, China; ^3^ Research Center for Osmanthus fragrans, Xianning Research Academy of Industrial Technology of Osmanthus fragrans, Xianning, China; ^4^ Science Division, IGENEBOOK Biotechnology Co., Ltd., Wuhan, China; ^5^ College of Pharmacy, Hubei Universily of Science and Technology, Xianning, China

**Keywords:** sweet osmanthus (*Osmanthus fragrans*), ethylene-responsive transcription factor (ERF), organic acid decomposition metabolism, flower senescence, gene expression patterns

## Abstract

*Osmanthus fragrans* is an ethylene-sensitive flower, and flower senescence was mediated by ethylene-responsive transcription factors (*OfERFs*). A total of 227 *OfERFs* were identified from *O. fragrans*, which were classified into five subfamilies: AP2 (35), DREB (57), ERF (125), RAV (6), and Soloist (4). Gene composition and structural analysis indicate that members of different subfamilies have different gene structures and conserved domains. Their gene promoter contains various functional responsive elements, including auxin, jasmonic acid, and other responsive elements. Among them, 124 *OfAP2/ERF* genes have expressed at any stage of flowering, and 10 of them may play roles in flowering or senescence. By comparative transcriptome analysis, *OfAP2/ERFs* affected by ethephon (ETH) and 5′-azacytidine (Aza) treatment were divided into three categories, which have various target gene sets. Importantly, these target gene sets participate in similar or different biological processes and metabolic pathways, suggesting that ethylene and DNA hypomethylation have crosstalk and a unique mechanism in regulating the flower senescence of *O. fragrans.* Co-expression analysis revealed that several key *OfAP2/ERFs* played a central role in organic acid metabolism and biosynthesis of branched-chain amino acids (BcAAs), among which *OfERF017* was selected for further functional analysis. Overexpression of *OfERF017* leads to significant enrichment of genes in organic acid metabolism pathways, which leads to a decrease in organic acid levels and promoting the flower senescence of *O. fragrans*. Together, these results give insights into the characteristics and functional analysis of *OfAP2/ERF* genes in *O. fragrans.*

## 1 Introduction

Sweet osmanthus (*Osmanthus fragrans*) is an ornamental plant in the Osmanthus family. As one of the top 10 famous traditional flowers in China cultivated for more than 2,500 years, it has been widely used in streets or gardens (Shi et al., 2024; Yue et al., 2024). Because of its unique aroma, sweet osmanthus is also an important economic fragrant flower in China and widely used in food additives or skincare products (Pan et al., 2021; Wu et al., 2022; Zhou et al., 2024). However, the short flowering period with an optimal harvesting time of 2–3 days has greatly limited both its ornamental and economic value (Zou et al., 2014). Therefore, understanding the flower senescence mechanism of sweet osmanthus is important for the harvest utilization and improvement of industrial products.

Flower senescence in ornamental plants is often associated with endogenous ethylene climacteric induced by pollination (Luangsuwalai et al., 2011; Sun et al., 2021), while, economically, harvest cultivars of sweet osmanthus without fruits are also sensitive to ethylene and non-pollination-induced endogenous ethylene climacteric is an important regulator for flower senescence (Zhou et al., 2008; Zou et al., 2014). The visible flower senescence characteristics coincide with the peak of the endogenous ethylene production. Exogenous ethylene treatment has not only significantly accelerated the abscission and wilting of cut flower petals, but also led to the central vacuole rupture, cell deformation, DNA degradation, and membrane lipid peroxidation in *O. fragrans* (Zou et al., 2014, 2017). Therefore, ethylene is an important regulatory factor in the flower senescence of *O. fragrans*. It was also found that DNA methylation decreased during the flower opening and senescence of *O. fragrans*, and the number of ethylene-responsive transcription factors (ERFs) associated with hypomethylated differentially methylated regions (Hypo-DMRs) was significantly enriched, suggesting that DNA hypomethylation participates in the flower senescence mediated by *OfERF*s through the ethylene response pathways (Zou et al., 2023). However, the specific regulatory networks of *OfAP2/ERFs* on flower senescence are still unclear.

APETALA2/ethylene-responsive factor (AP2/ERF) are plant-specific transcription factors and play important roles in plant growth, development, maturation, and senescence, as well as biotic and abiotic stress processes (Feng et al., 2020). AP2/ERF family members contain an AP2/ERF domain composed of 60–70 amino acids. According to the number and type of structural domains, the AP2/ERF family can be divided into five subfamilies: AP2, ERF, DREB, Soloist, and RAV. Among them, members of the AP2 subfamily contain two AP2/ERF domains, which have important functions in the regulation of developmental processes (Zhang et al., 2021a). Members of the ERF, DREB, and Soloist subfamilies usually only have one AP2/ERF domain. The members of the RAV subfamily contain not only one AP2/ERF domain, but also one B3 domain, which are important in the signal transduction of plant hormones (Alonso et al., 2003; Li et al., 2022a). Based on the conserved amino acid and binding sequences, the ERF subfamily with the 14th and 19th amino acids alanine (A) and aspartic acid (D), respectively, specifically bind to the cis-acting element GCC box with a conserved sequence of AGCCGCC and participate in ethylene response and abiotic stress (Li et al., 2022b). The DREB subfamily with the 14th and 19th amino acids valine (V) and glutamate (E), respectively, specifically bind to dehydration-responsive elements (DRE)/C-repeat (CRT) elements and are involved in response to drought and cold stress (Sakuma et al., 2002; Yang et al., 2021). Thus, the systematic identification of *OfERFs* in *O. fragrans* will help in the further exploration of their response patterns to flower senescence.

Based on the genome of *O. fragrans* “Liuye Jingui”, the *OfAP2/ERFs* family was identified. Combined with the transcriptomic data for flowers in different developmental stages and cut flowers treated with 5′-azacytidine (Aza) and ethephon (ETH), we analyzed the ethylene response members in the *OfAP2/ERF* family, as well as their potential downstream regulatory networks. Then, the function of *OfERF017* in organic acid decomposition metabolism was characterized. These results not only provide a foundation for the further exploration of the biological functions of genes in the *OfAP2/ERF* family, but also provide clues for unraveling the ethylene regulatory mechanism in the flower senescence of sweet osmanthus.

## 2 Materials and methods

### 2.1 Plant materials

Plant tissues (root, stem, leaf, and flower) of *O. fragrans* “Liuye Jingui” was collected from the Huazhong Agricultural University campus (Wuhan, China) (30°29′N, 114°21′W). Flowers were collected at six flowering stages (S1–S6: bud stage, initial flowering stage, early full flowering stage, full flowering stage, late full flowering stage, and abscission stage) as described in previous studies (Chen et al., 2021; Zou et al., 2023). Samples were weighed and conserved in an ice box or frozen in liquid nitrogen immediately for testing or stored at −80°C until further analysis.

### 2.2 DNA methylation inhibitor and ethephon treatment

Detached branches with floral buds at the S1 stage (bud stage) were treated with 10 mM Aza and 50 mg L^−1^ ETH dissolved in distilled water. As a control, cut flowers were placed in individual dishes containing distilled water. After 24 h, all samples were transferred to distilled water and kept at 25°C under a 12 h photoperiod with cool-white fluorescent lighting at an intensity of 10 mol m^−2^ s^−1^, in accordance with a previous study (Zou et al., 2014).

### 2.3 Genome-wide identification and phylogenetic and synteny analysis of OfAP2/ERFs

To identify the *OfAP2/ERF* gene family members in the sweet osmanthus genome, the protein sequences of AtAP2/ERF in *Arabidopsis* were downloaded from the PlantTFDB (Plant Transcription Factor Database; https://planttfdb.gao-lab.org/). Potential OfAP2/ERFs were isolated from the genome of *O. fragrans* “Liuye Jingui” using SwissProt and ITAK annotations. Then, a Hidden Markov Model (HMM) of PF00847 (AP2 domain) and PF02362 (B3 domain) was performed from the Pfam database (http://pfam.sanger.ac.uk/) to obtain candidate members of the OfAP2/ERFs family (Finn et al., 2011; Xu et al., 2015; Chen et al., 2021).

For phylogenetic analysis, MEGA11 (Kumar et al., 2018) was used to investigate the phylogenetic interactions of *OfAP2/ERFs* between *O. fragrans* and *Arabidopsis*. The *Arabidopsis* and *O. fragrans Of*AP2/ERF protein sequences were aligned by the MUSCLE program with default parameters. The multiple sequence alignment generated was analyzed and then used to build phylogenetic trees with the neighbor-joining method (with 1,000 bootstrap replicates), and evolutionary distances were computed using Poisson correction with pairwise deletion.

Circos v0.63 (http://circos.ca/) was employed to investigate their tandem duplication and synteny relationships as previously described (Li et al., 2013; Fan et al., 2017). Tandem duplication OfAP2/ERF genes was identified according to their physical locations on individual chromosomes in the sweet osmanthus genome. Adjacent homologous OfAP2/ERF genes on the same sweet osmanthus chromosome with no more than one intervening gene were considered tandem duplicates. The synteny blocks among different sweet osmanthus chromosomes were identified using the mcscan software (Wang et al., 2012).

### 2.4 Gene structure, conserved motifs, and cis-acting element analysis

For the gene structural analysis, gene models were downloaded from the sweet osmanthus genome (Chen et al., 2021). They were then submitted into the Gene Structure Display Server (http://gsds.cbi.pku.edu.cn/) for structural analyses (Hu et al., 2015). Additionally, the OfAP2/ERF protein sequences were employed to investigate conserved motifs with the MEME Suite platform (http://meme-suite.org/), and 30 motifs were found within each gene family. Analysis of Gene Ontology (GO) terms was performed with the online database (http://www.geneontology.org).

A sequence 2,000 bp upstream of the transcription start site of each *OfAP2/ERF* gene was selected to predict CREs, using PlantCARE (http://bioinformatics.psb.ugent.be/webtools/plantcare/html) (Magali et al., 2002; Xia et al., 2024).

### 2.5 Gene expression profile and the protein–protein interaction network

The genome and transcriptome sequencing data in different tissues (root, stem, and leaf) and nature flowering stages (S1–S6) of *O. fragrans* ‘Liuye Jingui’ are the same as those used in a previous study (NCBI accession number PRJNA679852) (Chen et al., 2021).

Cut flowers treated with 50 mg L^−1^ ETH, 10 mM Aza, and distilled water in different days were collected for RNA preparation. RNA sequencing libraries were sequenced and analyzed by IGENEBOOK Biotechnology Co. Ltd as previously described (Chen et al., 2021). The transcript levels were calculated with the fragments per kilobase of exon per million fragments mapped (FPKM) method. The transcriptome sequencing data using different treatments—50 mg L^−1^ ETH, 10 mM Aza, and distilled water—have been deposited into the NCBI BioProject under the accession number PRJNA798559.

The STRING database (v12.0) was used to construct a protein–protein interaction (PPI) network (Szklarczyk et al., 2023). Based on the whole genome sequence and transcriptome data, 124 *OfAP2/ERF* members of *O. fragrans* were used to predict the PPI network. The minimum required interaction score was set to medium confidence (0.4). Cytoscape (v3.10.0) was used for image processing (Shannon et al., 2003).

### 2.6 Promoter acquisition and downstream gene analysis of *OfAP2/ERF*

TBtools (v2.052) was used to extract promoter sequences from the genome of *O. fragrans* ‘Liuye Jingui’ (Chen et al., 2023). Using the FIMO program (v5.5.5), genes containing GCCGCC (GCC box) and G/A (R) CCGAC (DRE/CRT core) were scanned from the whole genome promoter, with a match *p*-value of 0.001.

### 2.7 Functional characterization of OfERF017 in transgenic *O. fragrans*

Flowers at the initial flowering stage (S2) of *O. fragrans* ‘Sijigui’ were collected for the transgenic experiment. Full-length *OfERF017* cloned into the pCAMBIA2300s vector and the empty pCAMBIA2300s vector was used as the negative control. The transgenic conditions are the same as those in a previous study (Xi et al., 2021). Three days after the inoculation, the flowers were collected for qRT-PCR and transcriptome sequencing analysis.

qRT-PCR was conducted utilizing Applied Biosystems 7500 sequence detection system (ABI7500; Thermo Fisher Scientific, Inc., Waltham, MA, USA). The experimental conditions and methods for qRT-PCR are the same as those used in previous studies, and the expression level of *Actin* served as the reference (Chen et al., 2021; Xia et al., 2024). The primers for qRT-PCR and full-length *OfERF017* were designed using Prime Premier 5 (**Supplementary Table S1**).

### 2.8 Ethylene production and quantification of widely targeted metabolites

Endogenous ethylene production was measured as previously described (Zou et al., 2023). Cut flowers were treated with Aza, ETH, and ddH_2_O, and flowers of transgenic *O. fragrans* were analyzed for ethylene production using a gas chromatograph (Agilent Technologies 7890B). The concentration was expressed as nanograms of ethylene per kilogram of plant fresh weight per second (ng kg^−1^ s^−1^).

Widely targeted metabolites were quantified as previously described (Chen et al., 2013). The widely targeted metabolites of flowers during different stages (S1–S6) and transgenic *O. fragrans* were quantified by MetWare (Wuhan, China) using the ultraperformance liquid chromatography–tandem mass spectrometry (UPLC-MS/MS) platform following standard procedures.

### 2.9 Data analysis

Three replicates were performed per treatment in each experiment. All the data are presented as means ± standard errors. Significant differences were obtained using SPSS with Duncan’s test at *p* < 0.05.

## 3 Results

### 3.1 Identification of OfAP2/ERFs in *O. fragrans*

**Figure 1:**
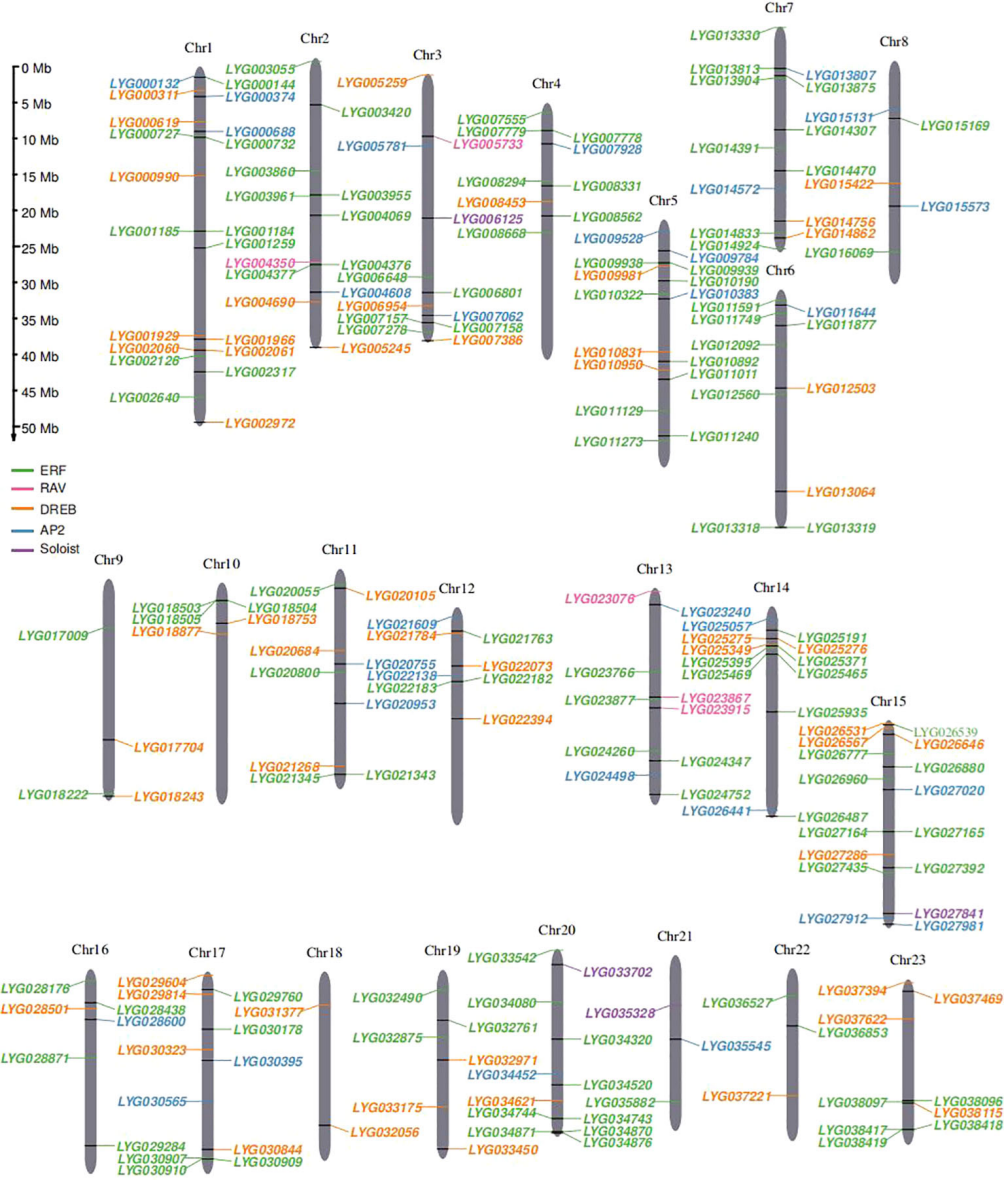
Chromosomal distribution of 227 *OfAP2/ERFs* genes on 23 *O. fragrans* chromosomes. The size of each chromosome and its corresponding *OfAP2/ERFs* gene distribution. Chr1–Chr23 are abbreviations for chromosome numbers 1–23, and black rectangles refer to gene pairs of tandem duplications.

In this study, a total of 227 *OfAP2/ERFs* genes were identified in the *O. fragrans* ‘Liuye Jingui’ genome according to the gene annotation and AP2 domain (PF00847) information. The protein sequences of these *OfAP2/ERFs* genes were extracted and compared with 172 AtAP2/ERF proteins in *Arabidopsis*. Then, the physical and chemical characterization of OfAP2/ERFs with deduced amino acid sequence was analyzed using ProtParam. The gene length of *OfAP2/ERF* ranged from 100 to 640 aa, the protein molecular weight (MW) ranged from 11.74 to 71.28 kDa, and the isoelectric point (pI) ranged from 4.24 to 9.8 (**Supplementary Table S2**). Among the 227 *OfAP2/ERF* members, 212 genes were successfully anchored to 23 chromosomes. From the distribution of *OfERF* genes, some of them displayed close locations from each other (**Figure 1**), which imply that the tandem duplication events might have occurred in this gene family.

**Figure 2:**
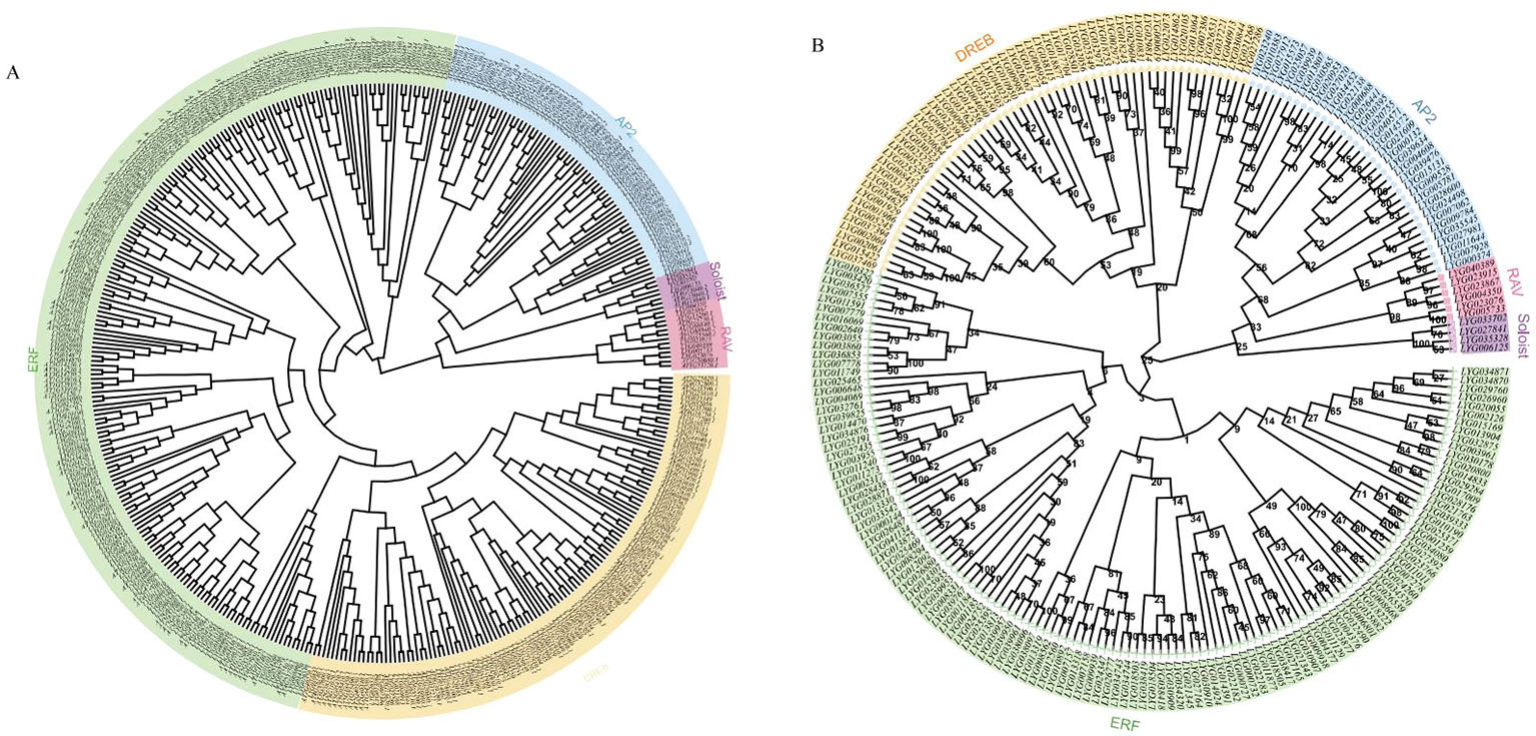
Phylogenetic analysis of OfAP2/ERFs in *O. fragrans* and *Arabidopsis*. **(A)** Phylogenetic analysis of OfAP2/ERFs in *O. fragrans* and *Arabidopsis*. **(B)** Phylogenetic analysis of the subfamily of OfAP2/ERFs genes.

In order to investigate the phylogenetic relationship between *O. fragrans* and *Arabidopsis*, multiple alignment analysis of 227 OfAP2/ERF proteins from *O. fragrans* and 172 AtAP2/ERF proteins from *Arabidopsis* protein was performed through conserved amino acid sequences obtained from the PlantTFDB (v5.0) (**Figure 2A**). The phylogenetic tree analysis has shown that each *OfAP2/ERFs* in *O. fragrans* can be found as a homologous *AtAP2/ERF* in *Arabidopsis* (**Figure 2A**). Based on the number of AP2 domains and sequence similarity, the 227 OfAP2/ERFs in *O. fragrans* are divided into five categories: the AP2 subfamily (35 members) that contains two AP2/ERF domains, the DREB subfamily (57 members), the ERF subfamily (125 members), the RAV subfamily (6 members) that contains one AP2/ERF domain and one B3 domain, and Soloist (4 members) (**Figure 2B**).

Gene duplication events play key roles in the evolution of a gene family. On the genome chromosomes of *O. fragrans*, there are a total of 160 pairs of genes exhibiting collinearity (**Supplementary Figure S1A**). These genes belong to the subfamilies AP2, DREB, ERF, RAV, and Soloist, with respective counts of 19, 43, 92, 1, and 5. Additionally, in interspecific collinearity analysis, there are 100 OfAP2/ERFs, forming 158 pairs of collinear gene pairs with genes in *Arabidopsis thaliana* (**Supplementary Figure S1B**). Among these, there are 61 ERF subfamily genes, 31 DREB subfamily genes, and 4 AP2 subfamily genes (LYG015131, LYG035545, LYG000374, and LYG022138), as well as 2 genes each in RAV (LYG023867 and LYG004350) and Soloist (LYG027841 and LYG033702). This indicates that the AP2/ERFs of *O. fragrans* have undergone gene expansion.

The average number of introns in all OfAP2/ERF genes is 1.5, ranging from 0 to 9, while the number of coding sequences (CDS) ranges from 1 to 10 (**Supplementary Figure S2**). Among them, LYG020953 belonging to the AP2 subfamily has the highest number of CDS, with 10. In different subfamilies, AP2 and Soloist genes have relatively more introns and CDS, averaging 5.3 and 6.3, and 5.8 and 5.8, respectively (**Supplementary Figures S2B, C**). In contrast, other subfamily genes mostly contain 0–2 introns and 1–2 CDS, with exceptions such as LYG005733 in RAV containing 5 introns (**Supplementary Figure S2A**), and LYG010322, LYG010892, and LYG013319 in ERF containing 3–5 introns and CDS (**Supplementary Figure S2E**). Many genes in the DREB subfamily do not even contain introns (**Supplementary Figure S2D**). The genes with less intron could have a rapid transcription and response to environmental change, which suggested a high-efficiency response of these *OfAP2/ERFs* to external threats.

Conserved motif analyses could imply functional differences among the family members. Among the 30 different motifs identified, motifs 1, 2, and 4 are present in all subfamilies. All genes contain one to nine different motifs, with the maximum observed in genes LYG021609 and LYG022138, and the minimum in LYG013330, which belongs to the ERF subfamily (**Supplementary Figure S2**). Specifically, the AP2 subfamily contains 10 different motifs, namely, motifs 1, 2, 3, 4, 6, 9, 12, 13, 25, and 26, with the first three being shared among all AP2 genes (**Supplementary Figure S2C**). The number of motifs in ERF subfamily genes ranges from 1 to 6, with a total of 17 different motifs (**Supplementary Figure S2E**). Motifs 1, 2, 3, and 4 are present in most genes, with motif 1 being shared among all genes except LYG013330. In the DREB subfamily, there are 12 different motifs, with motifs 1 and 2 being shared by all genes (**Supplementary Figure S2D**). In the RAV and Soloist subfamilies, motifs 3, 4, 11, and 14 are conserved in eight different motifs in RAV, while motif 21 is a conserved motif specific to the Soloist subfamily among its four motifs (**Supplementary Figures S2A, B**). Most of the genes in the same subfamily showed similar motif components.

To reveal the underlying regulatory mechanism of *OfAP2/ERFs*, cis-elements of *OfAP2/ERFs* were analyzed. The elements are mainly categorized into four major classes: growth and development, light, hormones (auxin, gibberellin, salicylic, abscisic, and MeJA), and stress response (drought, temperature, anaerobic, etc.), with 355, 1,841, 2,901, and 768 elements, respectively (**Supplementary Figure S3**). There are differences in both the total number and types of elements contained among different subfamilies. The ERF and DREB subfamilies have a higher proportion of elements in all four major categories, while RAV and Soloists have fewer. Specifically, the ERF subfamily genes have the highest proportion of elements in the light response category, reaching 55.57%, followed by DREB (34.75%). The number of elements in different genes ranges from 11 (LYG025191) to 44 (LYG007779). In terms of light response elements, genes in the ERF subfamily, such as LYG026539 (27), LYG032056 (26), and LYG029604 (25), have relatively more elements, while LYG020755 has the fewest, with only 4 elements. In hormone response elements, the gene LYG029284, also belonging to the ERF subfamily, has the most elements (24), while the gene LYG024347 has only 1 element. Additionally, all these elements comprise 62 different types, with the light response category having the highest number of types, with 34, of which the DREB subfamily has the most types (31), followed by ERF. These findings indicated that OfAP2/ERFs might play roles in a variety of pathways.

### 3.2 Expression patterns of *OfAP2/ERFs* in tissues and treatments

**Figure 3:**
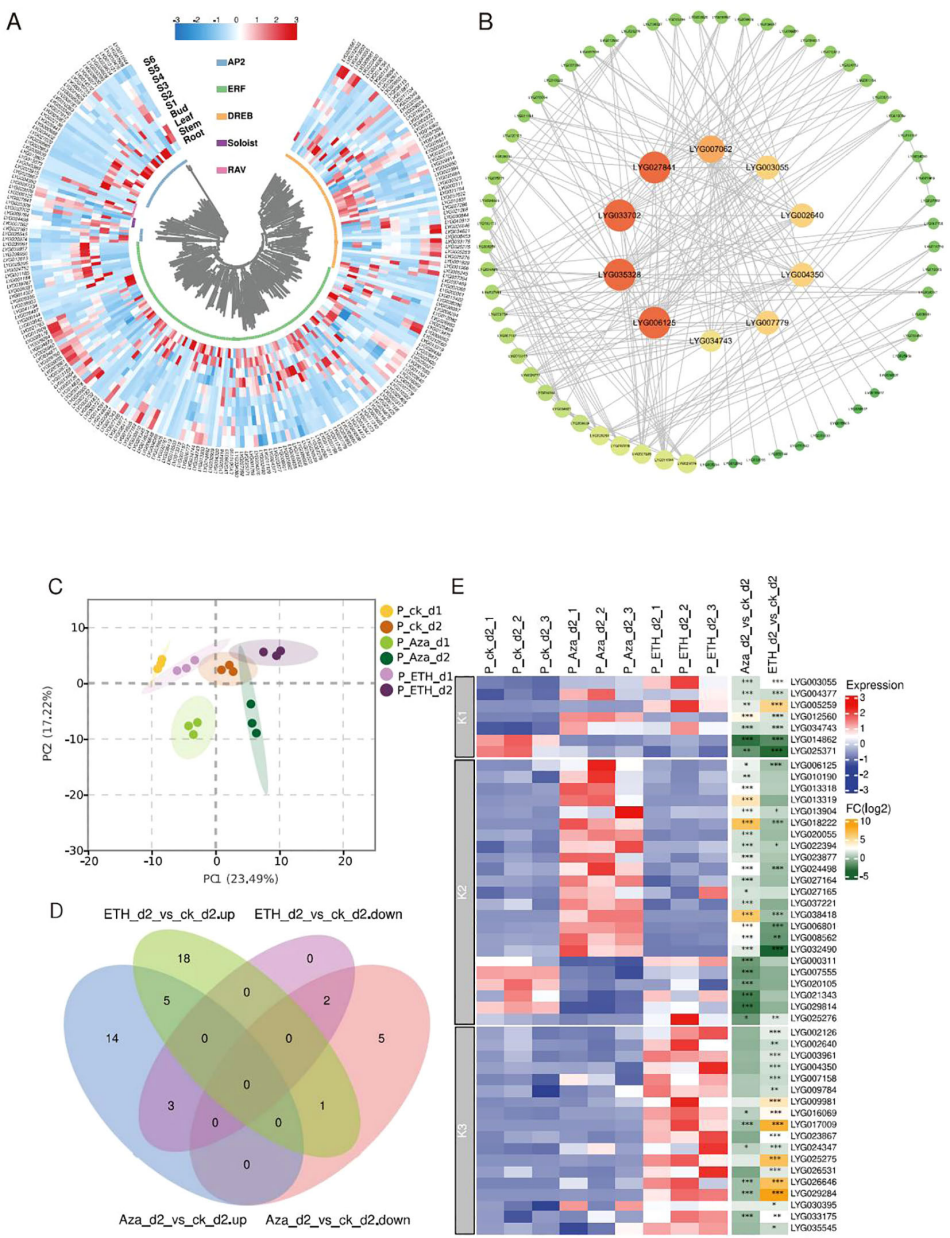
Expression profile and protein–protein interaction of OfAP2/ERFs. **(A)** Heatmap showing the transcript level of *OfAP2/ERFs* in the root, stem, leaf, and flowering stages (S1 to S6). **(B)** Protein–protein interaction. The orange and big circles represent more interaction pairs, and green and small circles represent fewer interaction pairs. **(C)** The principal component analysis (PCA) of the *OfAP2/ERFs* expression level under ETH and Aza treatment. **(D)** Venn diagram of the up- and downregulated genes in ETH_d2 or Aza_d2 relative to ck_d2, respectively. **(E)** Heatmap showing the transcript level of *OfAP2/ERFs* under ETH and Aza treatment and the fold change in ETH_d2_vs_ ck_d2 and Aza_d2_vs_ ck_d2 comparison.

Gene expression patterns in various tissues can give clues to predict gene function. Using RNA-seq, we analyzed the expression profile of *OfAP2/ERFs* in different tissues (root, stem, and leaf) and flowering stages (S1 to S6) of *O. fragrans*. Using FPKM > 1 as effective expression, it was found that the highest number of 124 *OfAP2/ERFs* is expressed in the flower [at least at any stage (S1 to S6)], followed by the root (109), stem (95), and leaf (79) (**Figure 3A**). These have indicated that *OfAP2/ERFs* may play important roles in flowers. In order to further investigate the key role that *OfAP2/ERFs* may play in flowers, we constructed a PPI network between 124 OfAP2/ERF members that are expressed in flowers. After filtering, 71 OfAP2/ERFs have been left in the network, among which LYG027841, LYG033702, LYG035328, LYG006125, LYG007062, LYG004350, LYG002640, LYG003055, LYG007779, and LYG034743 are at the central position, indicating their key roles in flowering (**Figure 3B**).

To investigate the similarities and differences of the response pattern of *OfAP2/ERFs* to ethylene in sweet osmanthus, we performed RNA-seq in cut flowers treated with 10 mM Aza and 50 mg L^−1^ ETH, both of which have caused earlier flower senescence and an increase of endogenous ethylene production (**Supplementary Figures S4A, B**). On day 1 and day 2, the endogenous ethylene production has increased to 39.20 and 31.33 ng^−1^ kg^−1^ s^−1^ under ETH treatment; the corresponding values are 6.29 and 5.29 ng^−1^ kg^−1^ s^−1^ under control (**Supplementary Figure S4B**). Correspondingly, Aza treatment increased the endogenous ethylene release to 16.98 ng^−1^ kg^−1^ s^−1^ on day 1 and to 14.49 ng^−1^ kg^−1^ s^−1^ on day 2 (**Supplementary Figure S4B**).

By the principal component analysis (PCA) of all genes, the samples have been only separated from each other treated by different days, but have not been separated from each other among different treatments (**Supplementary Figure S5A**). On the other hand, consistent with the increase in endogenous ethylene production, PCA shows that significant changes have occurred in ethylene biosynthesis genes and *OfAP2/ERFs* among the different treated samples (**Supplementary Figure S5B**; **Figure 3C**). A total of 15 and 24 *OfAP2/ERFs* have upregulated on day 1 and day 2 under ETH treatment, while 28 and 22 *OfAP2/ERFs* have upregulated on day 1 and day 2 under Aza treatment. The differentially expressed *OfAP2/ERFs* members on day 2 were used to perform the Venn analysis (**Figure 3D**). The results showed that the expression pattern of seven *OfAP2/ERF* members was consistent under ETH and Aza treatment, which were defined as Aza-mediated ethylene-dependent response factors (K1-ERFs). Four *OfAP2/ERFs* showed opposite changes under ETH and Aza treatments, while 19 *OfAP2/ERFs* were only affected by Aza treatments. These 23 genes are defined as Aza-dependent response factors (K2-ERFs). Additionally, 18 *OfAP2/ERFs* were only affected by ETH treatment and were defined as ethylene-dependent response factors (K3-ERFs). The heatmap clearly displays the expression patterns and variation relationships of these three types of *OfAP2/ERFs* (**Figure 3E**).

### 3.3 Downstream gene analyses of three types of *OfAP2/ERFs*

**Figure 4:**
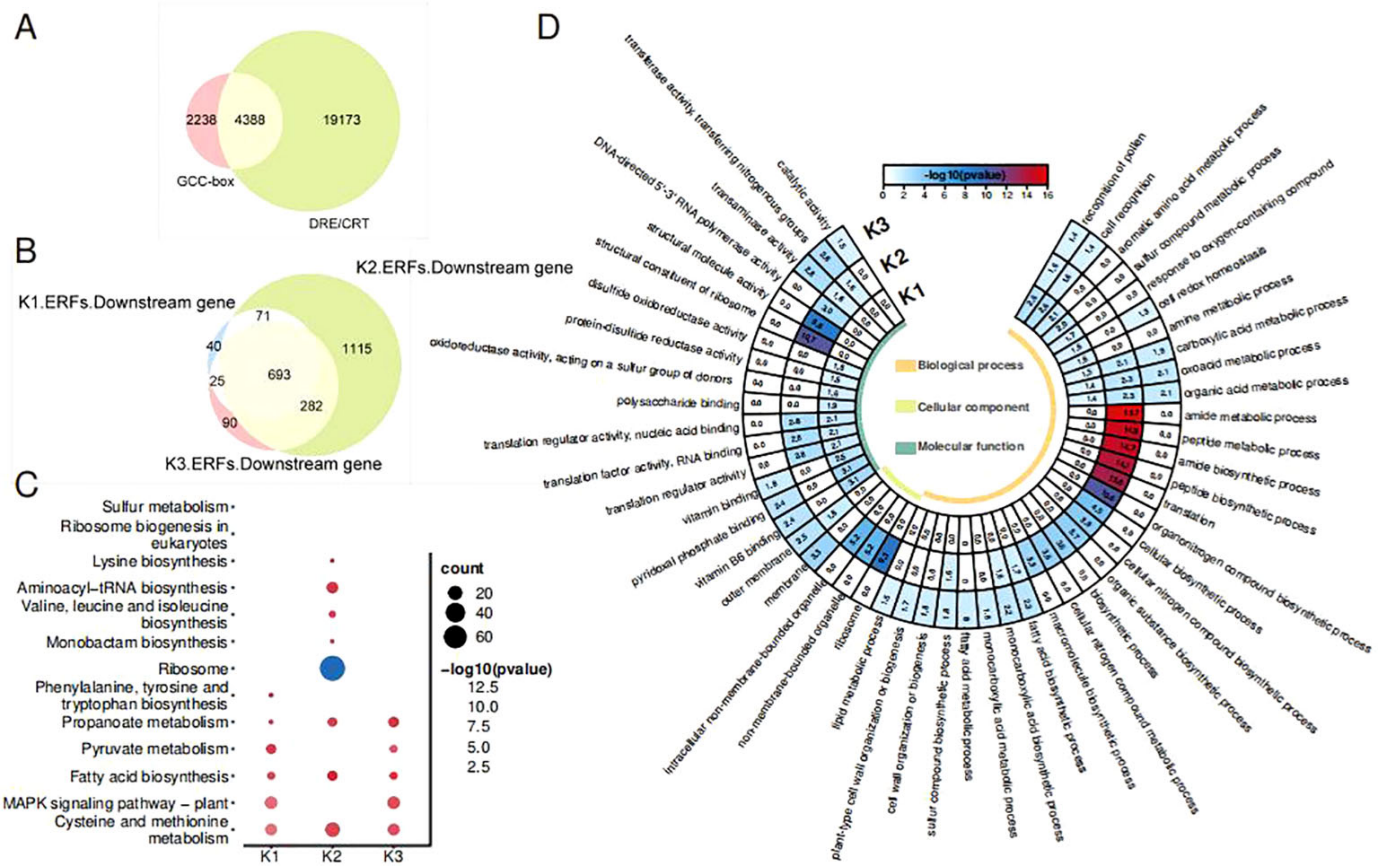
*OfAP2/ERF* downstream gene analysis. **(A)** Venn diagram of the promoter includes the GCC box or DRE/CRT genes. **(B)** Venn diagram of the three cluster ERF downstream genes. **(C)** GO analysis of the three types of ERF downstream genes. **(D)** KEGG analysis of the three types of ERF downstream genes.

As the ETH and Aza treatments have led to a different expression of *OfAP2/ERFs*, the downstream target genes of three types of *OfAP2/ERFs* were analyzed. The ERF protein binds to target genes by recognizing the GCC box or DRE/CRT cis-elements in the promoter. We scanned 6,626 promoter genes containing the GCC box and 23,561 promoter genes containing DRE/CRT from the genome of *O. fragrans* ‘Liuye Jingui’, respectively. The Venn plot shows that 4,388 gene promoters contain both the GCC box and DRE/CRT cis-elements (**Figure 4A**), which were used for further analysis. A total of 829 downstream genes of K1-ERFs, 2,161 downstream genes of K2-ERFs, and 1,090 downstream genes of K3-ERFs were obtained after felting with a threshold |cor| > 0.8 and *p* < 0.05 (**Figure 4B**). The downstream target genes of the three types of ERFs have overlapped in a considerable proportion (693/2,316), indicating that Aza and ethylene have similar pathways in regulating flower senescence in *O. fragrans*. Compared to the K1-ERFs and K3-ERFs groups, the downstream target genes in the K2-ERFs group have the most unique genes, suggesting a special regulatory pathway for DNA hypomethylation to regulate downstream gene function through *OfAP2/ERFs*.

To further investigate the pathways regulated by *OfAP2/ERFs* under ETH and Aza treatment, we conducted GO and KEGG enrichment analysis on downstream target genes of three types of OfERFs. GO enrichment has shown that downstream genes were involved in pollen recognition, cell recognition, the carboxylic acid metabolic process, the oxoacid metabolic process, and the organic acid metabolic process (**Figure 4C**). However, downstream genes of the K2-ERFs group are involved in more unique biological processes, such as translation, amide and peptide biosynthetic and metabolic processes, and organic and cellular nitrogen compound biosynthetic processes.

According to the KEGG enrichment, downstream genes of all three types of *OfERFs* are involved in cysteine and methionine metabolism, propanoate metabolism, and fatty acid biosynthesis (**Figure 4D**). Additionally, downstream genes of the K1-ERFs and K3-ERFs groups are involved in the MAPK signaling pathway plant, which is known to be associated with ethylene signaling transduction (Xu and Zhang, 2014; Chen et al., 2017). However, the downstream genes of the K2-ERFs group are independently involved in aminoacyl-tRNA biosynthesis and valine, leucine, and isoleucine biosynthesis. A recent study has shown that CHH hypermethylation induces petal senescence in carnations by reducing the accumulation of branched-chain amino acids (BcAAs, including valine, leucine, and isoleucine) (Feng et al., 2024). These results indicate that the Aza treatment-mediated DNA demethylation affects the decomposition metabolism of carboxylic acids, oxygen-containing acids, and organic acids, as well as the biosynthesis of fatty acids, through the ethylene signaling pathway. On the other hand, independent ethylene signaling pathways affect nitrogen metabolism, amide tRNA biosynthesis, and BcAA biosynthesis, mediating the flowering and senescence of sweet osmanthus.

### 3.4 Transcriptional regulatory network of organic acid metabolism and BcAA biosynthesis

**Figure 5:**
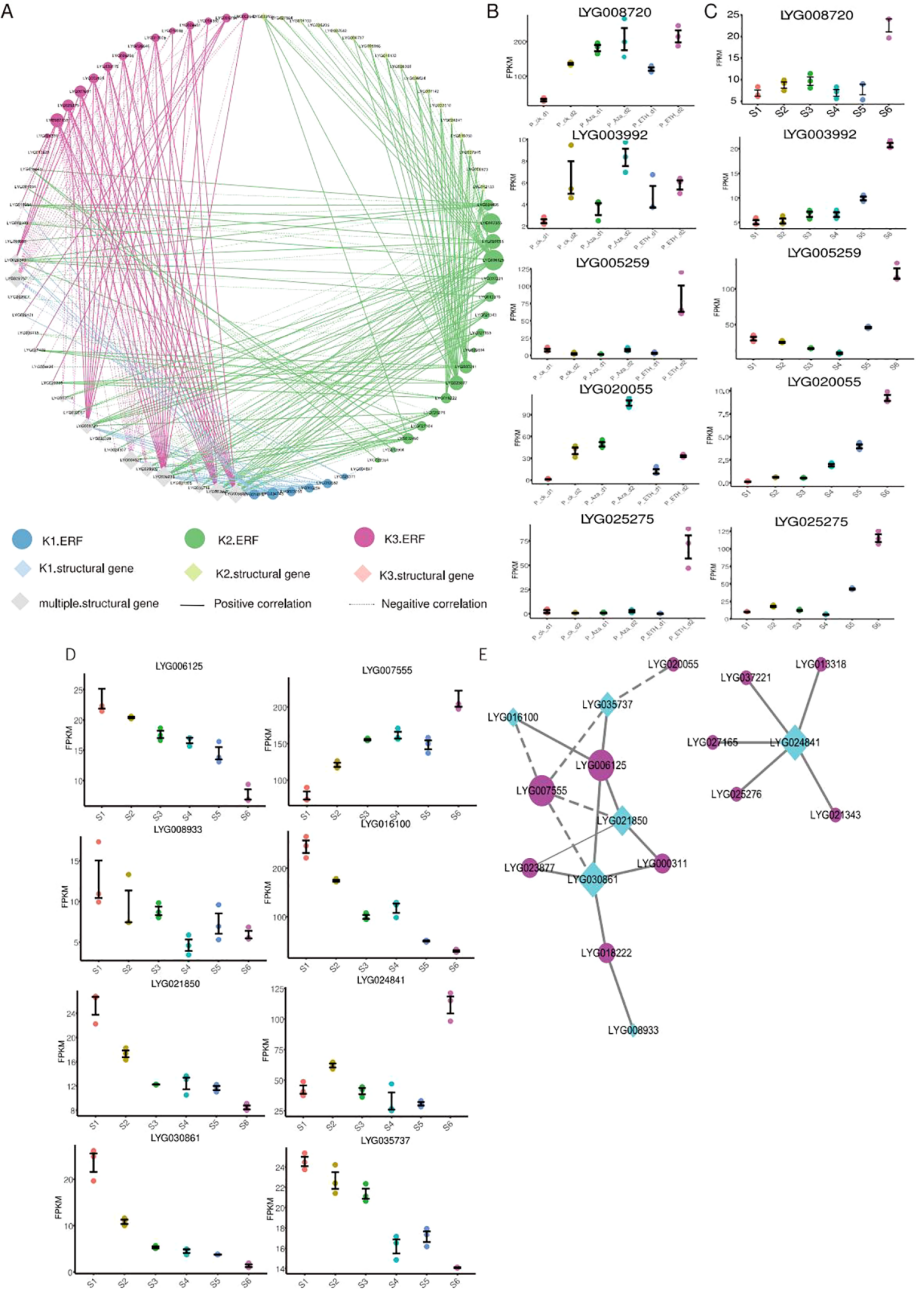
Analysis of genes involved in organic acid metabolism and branched-chain amino acid biosynthesis. **(A)** Transcriptional regulatory network of the organic acid metabolism process. The ‘multiple structural gene’ represent these genes are downstream cluster of ERFs.. **(B)** Expression of key genes involved in organic acid metabolism at six flowering stages. **(C)** Expression of key genes involved in organic acid metabolism under ETH and Aza treatment. **(D)** Expression of key genes involved in branched-chain amino acid biosynthesis at six flowering stages. **(E)** Transcriptional regulatory network of branched-chain amino acid biosynthesis.

Because of the GO enrichment analysis of downstream genes in K1-ERF, K2-ERF, and K3-ERF types, which simultaneously point to organic acid metabolism pathways (**Figure 4C**), we then constructed a transcriptional regulatory network for organic acid metabolism (**Figure 5A**). It regulates a total of 46 organic acid metabolism genes, of which 15 are simultaneously regulated by all three types of *OfERFs*, suggesting their similar functions. K1-ERFs regulate 18 organic acid metabolism genes, of which 16 are also regulated by K3-ERFs. Additionally, K2-ERFs independently regulate 19 organic acid metabolism genes. These results indicate that the flower senescence induced by ETH or Aza may both be achieved through the organic acid metabolism pathway mediated by *OfERFs*.

In the whole transcriptional regulatory network, *LYG039238*, *LYG008720*, *LYG006602*, *LYG028609*, *LYG003992*, *LYG009757*, and *LYG004527* are the key genes of organic acid metabolism (**Figure 5A**), and three of them upregulated during the flower senescence of sweet osmanthus. Among them, *LYG008720* and *LYG003992* are also induced by Aza and ETH treatments (**Figures 5B, C**). *LYG034743*, *LYG014862*, *LYG005259*, *LYG006125*, *LYG020055*, *LYG000311*, *LYG007555*, *LYG023877*, *LYG003961*, *LYG007158*, and *LYG025275* are the key *OfERFs* in this network (**Figure 5A**), and most of them have upregulated during flower senescence. Among them, *LYG005259*, *LYG020055*, and *LYG025275* belong to the K1-ERFs, K2-ERFs, and K3-ERFs groups, respectively. *LYG005259* was induced by both Aza and ETH treatments, while *LYG020055* was induced by Aza treatment, and *LYG025275* was induced by ETH treatment (**Figures 5B, C**).

During the flower senescence of carnations, the accumulation of BcAAs decreases. Exogenous supplementation of BcAAs significantly delays the flower senescence of carnations (Feng et al., 2024), indicating that the decrease in BcAAs may be a hallmark of flower senescence. In our study, K2-ERFs regulated a total of six genes involved in BcAA biosynthesis. Interestingly, five of them downregulate within flower senescence (**Figure 5D**), which may lead to a decrease in the content of BcAAs during flower senescence. To understand the ERFs that regulate the BcAA biosynthesis, we constructed a transcriptional regulatory network from downstream genes of K2-ERFs (**Figure 5E**). The whole network can be divided into two sub-networks, one of which is composed of *LYG024841* and five ERFs, and the other sub-network consists of six ERFs and five BcAA synthase genes. *LYG030861* and *LYG021850* are the key BcAA synthase genes, while *LYG006125* and *LYG007555* are the key ERF genes. *LYG030861* is a homologous DHAD protein of *Arabidopsis*, which is almost not expressed during the abscission stage (S6 stage) of sweet osmanthus. LYG007555 encodes an ERF109 protein, which is upregulated during flower senescence (**Figure 5D**). In *Arabidopsis*, the mutation erf109 causes a decrease in ROS and delayed plant senescence (Lin et al., 2020). Silencing *BrERF109* in Chinese cabbage promotes flavonoid biosynthesis and delays leaf senescence (Yue et al., 2023). These results suggest that *LYG007555* may be a negative regulatory factor for flower senescence, delaying flower senescence by inhibiting the expression of *LYG030861* and reducing the content of BcAAs.

### 3.5 Functional characterization of *OfERF017* in transgenic *O. fragrans*

It was found that *OfERF017* (LYG025275) participates in the organic acid metabolism network, and its expression increases with flower senescence (**Figures 5A, C**), consistent with the increase of ethylene production (Zou et al., 2023) and the decrease of total organic acid in S1 to S6 (**Supplementary Table S3**). Thus, *OfERF017* was chosen as an example to investigate its function in organic acid decomposition metabolism and flower senescence.

**Figure 6:**
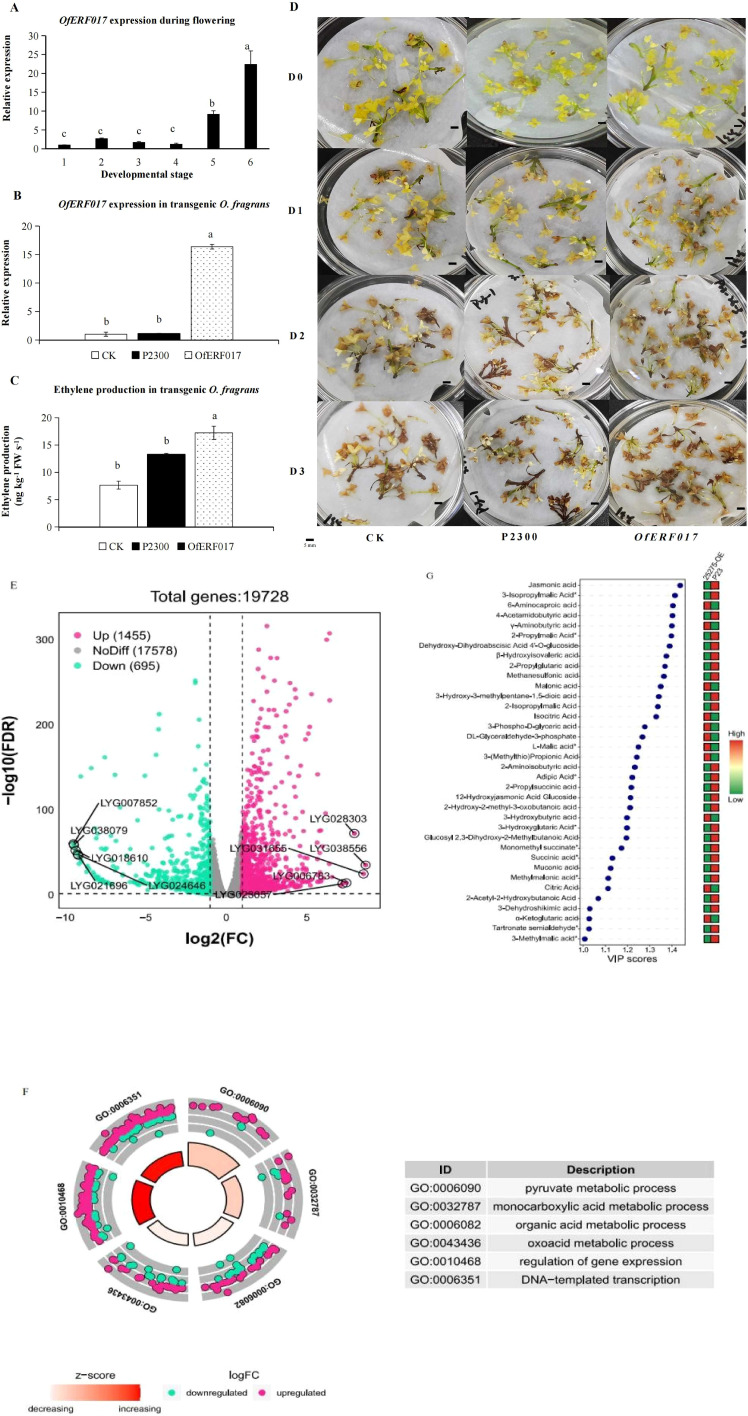
Functional characterization of *OfERF017* in transgenic *O. fragrans*. **(A)** The expression analysis of *OfERF017* during flowering. **(B)** The expression analysis of *OfERF017* in transgenic *O. fragrans.* **(C)** Ethylene production in *OfERF017*-overexpressing transgenic *O. fragrans.* **(D)** The phenotype of OfERF017-overexpressing transgenic flowers in *O. fragrans.* **(E)** Volcano map of differentially expressed genes (DEGs) in *OfERF017*-overexpressing transgenic flowers. **(F)** Gene Ontology enrichment results of DEGs in *OfERF017*-overexpressing transgenic flowers. **(G)** Heatmap of differential organic acid content in *OfERF017*-overexpressing transgenic flowers.

The results of qPCR showed that *OfERF017* was mainly expressed in S5 and S6 and reached its peak in S6 (**Figure 6A**). Overexpression of *OfERF017* in transgenic flowers of *O. fragrans* (*OfERF017*-OE) (**Figure 6B**) led to a significant increase of endogenous ethylene production (**Figure 6C**) and an earlier senescence phenotype (**Figure 6D**). After 1 day of inoculation, *OfERF017*-OE exhibited partial flower browning, with only a small number of flowers browning in CK and P2300. By the third day, all flowers of *OfERF017*-OE showed browning, while some flowers of WT and P2300 remained yellow without decay (**Figure 6D**). These results indicate that *OfERF017* promotes flower senescence in transgenic *O. fragrans*.

We then performed transcriptome analysis in *OfERF017*-OE and P2300 flowers. Based on |log2fold change| > 1 and FDR < 0.05, 2,150 differentially expressed genes (DEGs) were identified from *OfERF017*-OE, including 1,455 upregulated DEGs and 695 downregulated DEGs (**Figure 6E**; **Supplementary Table S4**). GO enrichment showed that these DEGs are involved in various biological processes (**Supplementary Table S5**). It is worth noting that the terms “DNA template transcription” and “regulation of gene expression” are significantly enriched (*p*-values of 9.89 × 10^−3^ and 4.69 × 10^−3^, respectively), representing the function of *OfERF017* as a transcription factor (**Figure 6F**). In addition, the “pyruvate metabolism process” (*p*-value of 1.62 × 10^−4^), “monocarboxylate metabolism process” (*p*-value of 1.05 × 10^−2^), “oxygenic acid metabolism process” (*p*-value of 3.88 × 10^−2^), and “organic acid metabolism process” (*p*-value of 2.79 × 10^−2^) were significantly enriched. It is interesting that the number of upregulated DEGs during these processes is greater than that of downregulated DEGs (**Figure 6F**). LC-MS/MS was used to detect organic acids in *OfERF017*-OE and P2300. A total of 65 organic acids were detected, with which the total content was downregulated from 2.00E+08 to 1.71E+08 in *OfERF017*-OE (**Figure 6G**). In summary, our research findings indicate that *OfERF017* responds to ethylene by inducing high expression of genes involved in organic acid metabolism pathways, leading to a decrease in organic acid levels and promoting the flower senescence of *O. fragrans*.

## 4 Discussion

Ethylene is an important regulatory factor in the flower senescence of *O. fragrans* (Zou et al., 2014), and DNA hypomethylation participates in the flower senescence mediated by *OfERF*s through the ethylene response pathways (Zou et al., 2023). In this study, a total of 227 *OfAP2/ERFs* genes were identified in the *O. fragrans* “Liuye Jingui” genome. Chromosome 1 contained the greatest numbers of *OfERFs* genes (20 *OfERFs*, 9.43%), while chromosome 18 contained the lowest numbers (only 2 *OfERFs*, 0.94%). There are only four *OfERFs* on chromosome 9 (1.89%) and three *OfERFs* on chromosomes 21 and 22 (1.42%). *OfERF* genes on other chromosomes are generally between 5 and 16 (84.9%) (**Figure 1**). It can be seen from this that the number of *OfERFs* distributed on each chromosome is not positively correlated with chromosome length. *OfAP2/ERFs* in *O. fragrans* are higher than those in most plant species, such as *A. thaliana* (172) (Sakuma et al., 2002), *Populus trichocarpa* (200) (Zhuang et al., 2008), *Hypericum perforatum* (101) (Zhang et al., 2021b), *Salix arbutifolia* (173) (Rao et al., 2015), *Camellia sinensis* (178) (Liu et al., 2023), *Rose chinensis* (137) (Li et al., 2020), and *Cucurbita moschia* (212) (Li et al., 2022a), and most of them have played an important role in abiotic stress. Especially in the ERF subfamily, there are 125 *OfERFs*, far exceeding 88 *SaERFs*, 88 *CsERFs*, and 92 *CmoERFs*. Additionally, collinearity analysis showed that the majority of *OfERF* gene pairs are segmental duplicates, with five pairs being tandem duplicates. Segmental duplicates generate homologous genes, and tandem duplicates produce gene clusters (Zhang and Li, 2018), which leads to an expansion in the number of *OfAP2/ERFs* genes.

To investigate the similarities and differences of the response pattern of *OfAP2/ERFs* to ethylene in sweet osmanthus, RNA-seq of cut flowers treated with Aza and ETH was performed, as both of them caused earlier flower senescence and an increase in endogenous ethylene production. According to the different expression patterns of *OfAP2/ERFs* in treatments, *OfAP2/ERFs* were divided into K1 to K3 groups, and the downstream target genes of the three types of *OfAP2/ERFs* were analyzed. GO enrichment analysis has shown that downstream genes were involved in pollen recognition, cell recognition, the carboxylic acid metabolic process, the oxoacid metabolic process, and the organic acid metabolic process (**Figure 4C**). Organic acids widely exist in plants and degrade during the development and storage of fruits and flowers (Zhang et al., 2014). It has been reported that nitrogen nutrition is closely related to ethylene signaling and plant senescence, and low nitrogen conditions can induce early leaf senescence in *Arabidopsis* (Cheng et al., 2023; Fan et al., 2023; Ma et al., 2023).

Citric acid, humic acid, and salicylic acid in organic acids are often used as preservatives for fresh cut flowers (Fan et al., 2015; Aziz et al., 2020; Rahbar et al., 2023), which cleared the ROS accumulated by senescent damage or by retaining cellular water. We constructed a transcriptional regulatory network for organic acid metabolism (**Figure 5A**). Among them, LYG008720 is a homologous protein of pyruvate kinase (PK) in *Arabidopsis*, which plays an important role in cellular senescence metabolic reprogramming (Dou et al., 2023; Wu et al., 2023). PKs are closely related to growth, development, and stress resistance in plants (Wulfert et al., 2020). LYG003992 is a homologous protein of sphingosine-1-phosphate lyase in *Arabidopsis*, involved in regulating the levels of long-chain bases and their phosphorylated derivatives (LCB-P), and may play a role in senescence of *Arabidopsis* (Chen et al., 2009). At the same time, the protein also plays a role in water deficiency stress (Nakagawa et al., 2012), which is in concordance with water loss during the flower senescence of *O. fragrans*. *LYG005259, LYG020055*, and *LYG025275* are the key *OfERFs*. *LYG005259* and *LYG025275* are homologous proteins of ERF017 in *Arabidopsis* that have been associated with nitrogen metabolism (Gaudinier et al., 2018) and fruit peel degreening (Han et al., 2018). *LYG020055* is a homologous protein of AtERF114 in *Arabidopsis* that is involved in nitrogen metabolism (Gaudinier et al., 2018) and disease resistance (Li et al., 2022b). Then, *LYG025275* (*OfERF017*) was chosen as an example to investigate its function in organic acid decomposition metabolism and flower senescence via transgenic flowers of *O. fragrans*. The results showed that overexpression of *OfERF017* induced the high expression of genes involved in organic acid metabolism pathways, leading to a decrease in organic acid levels and promoting the flower senescence of *O. fragrans*, which confirms the previous analysis that *OfAP2/ERFs* may regulate the flower senescence of *O. fragrans* through organic acid metabolism.

## 5 Conclusion

This is the first comprehensive analysis of the AP2/ERF superfamily in *O. fragrans* ‘Liuye Jingui’. The phylogenetic relationship, gene structures, protein interaction network, and expression patterns in various tissues and flowering stages were analyzed. By comparative transcriptome analysis, the downstream genes of *OfAP2/ERFs* affected by ETH and Aza treatment were analyzed, and these target gene sets participate in similar or different biological processes and metabolic pathways, suggesting that ethylene and DNA hypomethylation have crosstalk and a unique mechanism in regulating the flower senescence of *O. fragrans.* Co-expression analysis revealed that several key *OfAP2/ERFs* played a central role in organic acid metabolism and biosynthesis of BcAAs, among which *OfERF017* was selected for further functional analysis. Overexpression of *OfERF017* increased the endogenous ethylene production, leading to an earlier senescence phenotype and a decrease in organic acid levels and promoting the flower senescence of *O. fragrans.* Overall, these findings will lay a valuable foundation to elucidate the functions of *OfAP2/ERFs* in *O. fragrans*.

## Data Availability

The original contributions presented in the study are included in the article/**Supplementary Material**. Further inquiries can be directed to the corresponding authors.

## Supplementary material

Supplementary Figure 1 Synteny analysis of OfAP2/ERFs in *O. fragrans* and *Arabidopsis*. **(A)** Collinearity analysis of 227 *OfAP2/ERF*s in *O. fragrans*. The gray lines represent collinearity among all gene pairs in the *O. fragrans* genome, while the red lines indicate collinearity relationships between genes of the *OfAP2/ERF*s family. **(B)** Collinearity analysis between 172 *OfAP2/ERF*s of *O. fragrans* and *Arabidopsis* genes. The gray lines denote all collinear gene pairs between the *O. fragrans* and *Arabidopsis* genomes, whereas the purple lines represent the *OfAP2/ERF*s genes of *O. fragrans*. Genes labeled with different colors represent distinct subfamilies of *OfAP2/ERF*s.

Supplementary Figure 2 Gene sequence and motif analysis of OfAP2/ERF in sweet osmanthus. **(A)** RAV subfamily; **(B)** Soloist subfamily; **(C)** AP2 subfamily; **(D)** DREB subfamily; and **(E)** ERF subfamily. To reveal the underlying regulatory mechanism of *OfAP2/ERFs*, cis-elements of *OfAP2/ERFs*.

Supplementary Figure 3 Analysis of cis-elements in the promoters of *OfAp2/ERF* genes. **(A)** AP2 subfamily; **(B)** DREB subfamily; **(C)** RAV subfamily; **(D)** Soloist subfamily; **(E)** ERF subfamily. For panels A to E, the heatmap on the left displays the number of four major categories of cis-elements on different genes, where deeper shades of red indicate a greater quantity represented, while greener shades indicate fewer. The middle plot illustrates the positional distribution of different cis-elements on each gene. The stacked bar chart on the right represents the quantity of different types of elements on each gene.

Supplementary Figure 4 An earlier appearance of senescence and increase of endogenous ethylene production is triggered by Aza and ETH treatment. **(A)** Morphology of cut flowers treated with 10 mM 5′-azacytidine (Aza), distilled water (CK), and 50 mg L^−1^ ethephon (ETH) showing that the senescence process was accelerated in response to Aza and ETH treatment. **(B)** Aza and ETH treatment significantly increased the release of endogenous ethylene production of cut flowers (D1 and D2). Error bars indicate the SE. Asterisks indicate the significance at *p* < 0.05. Three biological replicates under Aza or distilled water treatment were used. ns, nonsignificant.

Supplementary Figure 5 The principal component analysis (PCA) of all genes and ethylene biosynthesis genes. **(A)** The PCA of samples based on all genes only separate in different vase life but not separate from each treatment. **(B)** The PCA of samples based on ethylene biosynthesis genes separate in both different vase life and treatment.
