# Supplementary figures and images for: Comprehensive transcriptome analysis of AP2/ERFs in Osmanthus fragrans reveals the role of OfERF017-mediated organic acid metabolism pathway in flower senescence
Source: Front Plant Sci. 2024 Sep 26;15:1467232. doi: 10.3389/fpls.2024.1467232 (PMC11464312; doi:10.3389/fpls.2024.1467232)

A

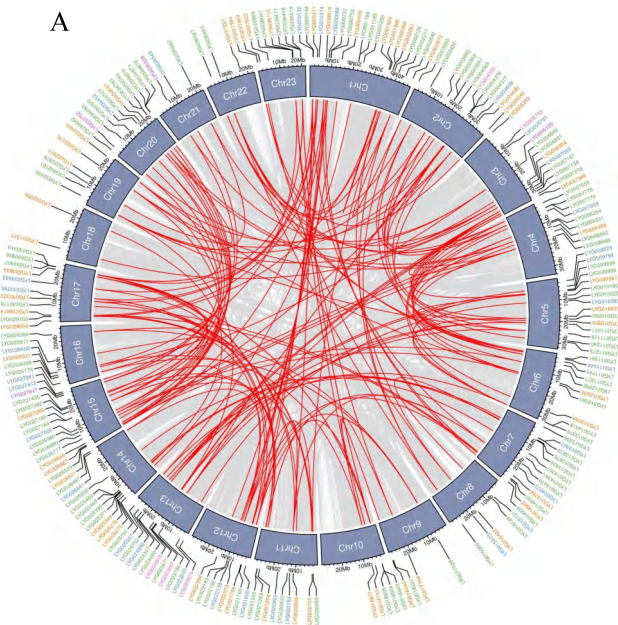

B

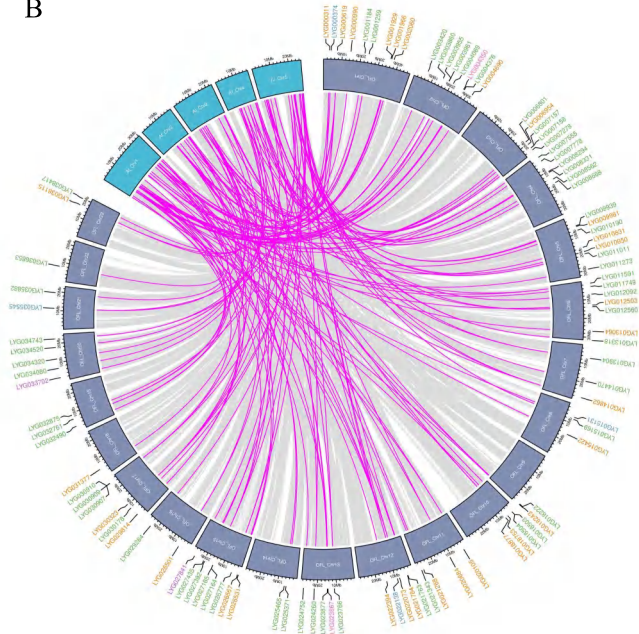

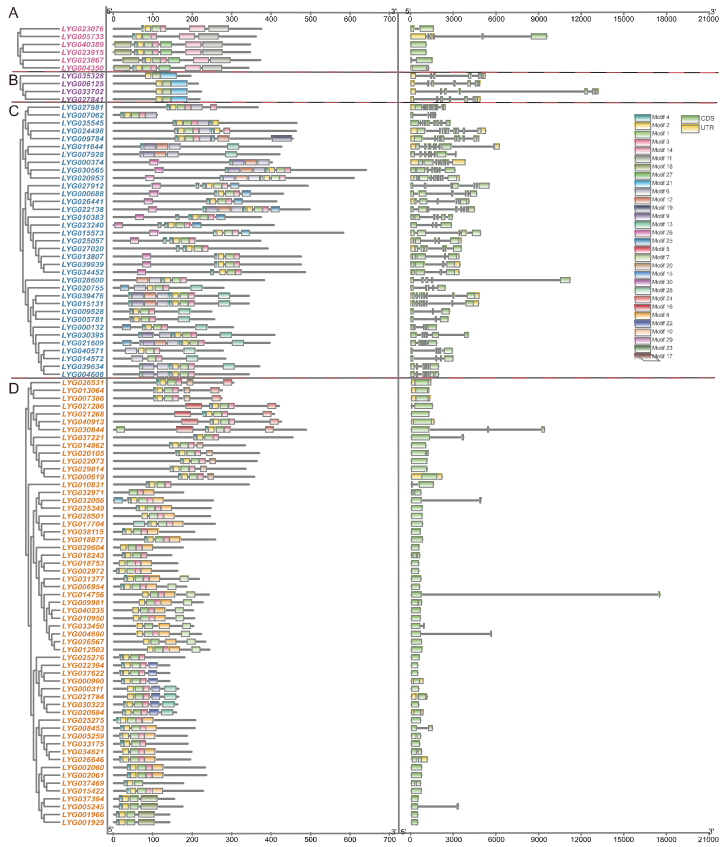

**E**

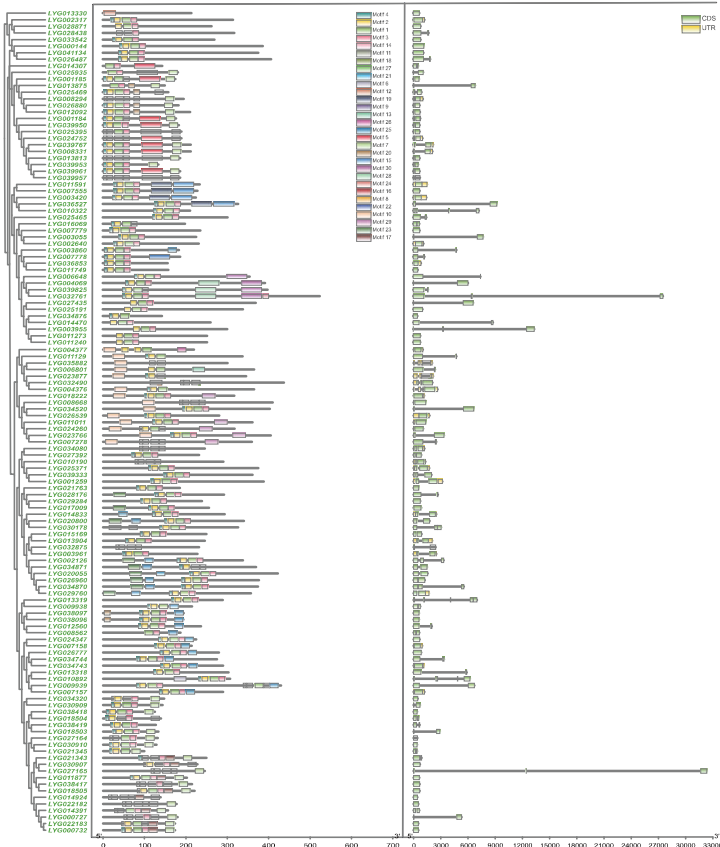



A

d0

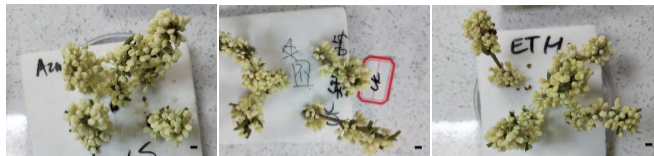

d1

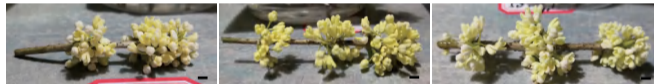

d2

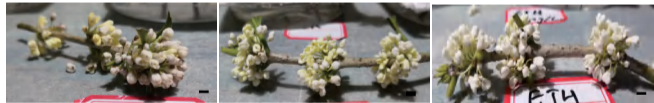

d3

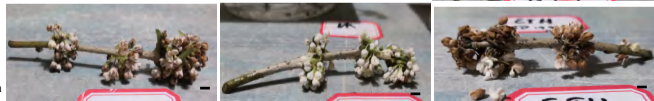

5 mm

B

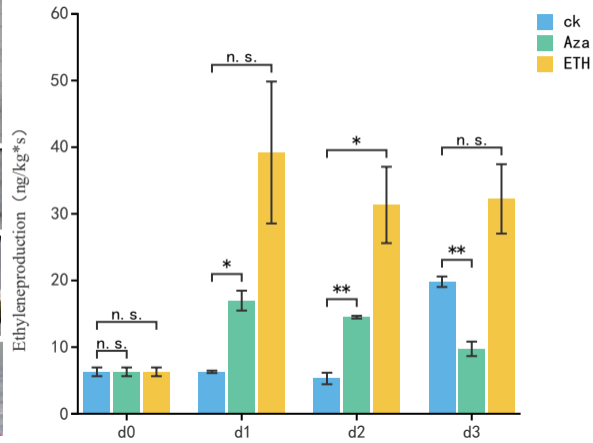

A

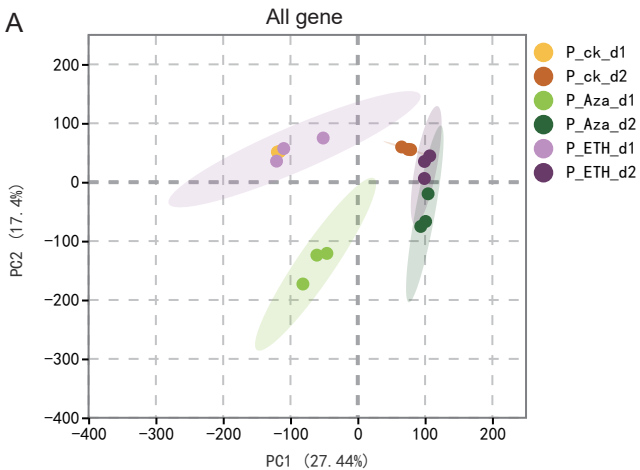

B

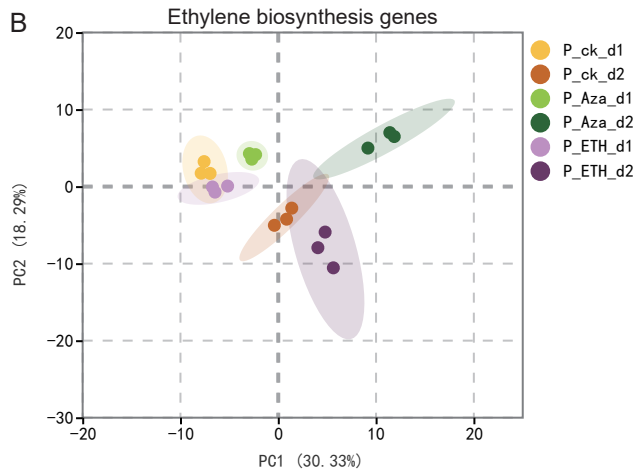

Supplement: Supplementary Figure 1 — Synteny analysis of OfAP2/ERFs in O. fragrans and Arabidopsis. (A) Collinearity analysis of 227 OfAP2/ERFs in O. fragrans. The gray lines represent collinearity among all gene pairs in the O. fragrans genome, while the red lines indicate collinearity relationships between genes of the OfAP2/ERFs family. (B) Collinearity analysis between 172 OfAP2/ERFs of O. fragrans and Arabidopsis genes. The gray lines denote all collinear gene pairs between the O. fragrans and Arabidopsis genomes, whereas the purple lines represent the OfAP2/ERFs genes of O. fragrans. Genes labeled with different colors represent distinct subfamilies of OfAP2/ERFs. [file DataSheet1.pdf]
